# BCL-XL is crucial for progression through the adenoma-to-carcinoma sequence of colorectal cancer

**DOI:** 10.1038/s41418-021-00816-w

**Published:** 2021-06-11

**Authors:** Prashanthi Ramesh, Tamsin R. M. Lannagan, Rene Jackstadt, Lidia Atencia Taboada, Nico Lansu, Pratyaksha Wirapati, Sander R. van Hooff, Danielle Dekker, Jessica Pritchard, Aleksandar B. Kirov, Sanne M. van Neerven, Sabine Tejpar, Geert J. P. L. Kops, Owen J. Sansom, Jan Paul Medema

**Affiliations:** 1 Laboratory for Experimental Oncology and Radiobiology, Center for Experimental and Molecular Medicine, AmsterdamUMC, University of Amsterdam, Cancer Center Amsterdam, Meibergdreef 9, 1105 AZ Amsterdam, The Netherlands; 2 Oncode Institute, Meibergdreef 9, 1105 AZ Amsterdam, The Netherlands; 3 Cancer Research UK Beatson Institute, Garscube Estate, Glasgow, G61 1BD UK; 4 Oncode Institute, Hubrecht Institute-KNAW and University Medical Center Utrecht, Utrecht, The Netherlands; 5 Swiss Institute of Bioinformatics, Lausanne, Switzerland; 6 Molecular Digestive Oncology, Department of Oncology, Katholieke Universiteit Leuven, Leuven, Belgium; 7 Institute of Cancer Sciences, University of Glasgow, Garscube Estate, Glasgow, G61 1QH UK

**Keywords:** Cancer models, Cancer stem cells, Cell biology

## Abstract

Evasion of apoptosis is a hallmark of cancer, which is frequently mediated by upregulation of the antiapoptotic BCL-2 family proteins. In colorectal cancer (CRC), previous work has highlighted differential antiapoptotic protein dependencies determined by the stage of the disease. While intestinal stem cells (ISCs) require BCL-2 for adenoma outgrowth and survival during transformation, ISC-specific *MCL1* deletion results in disturbed intestinal homeostasis, eventually contributing to tumorigenesis. Colon cancer stem cells (CSCs), however, no longer require BCL-2 and depend mainly on BCL-XL for their survival. We therefore hypothesized that a shift in antiapoptotic protein reliance occurs in ISCs as the disease progresses from normal to adenoma to carcinoma. By targeting antiapoptotic proteins with specific BH3 mimetics in organoid models of CRC progression, we found that BCL-2 is essential only during ISC transformation while MCL1 inhibition did not affect adenoma outgrowth. BCL-XL, on the other hand, was crucial for stem cell survival throughout the adenoma-to-carcinoma sequence. Furthermore, we identified that the limited window of BCL-2 reliance is a result of its downregulation by miR-17-5p, a microRNA that is upregulated upon *APC*-mutation driven transformation. Here we show that BCL-XL inhibition effectively impairs adenoma outgrowth in vivo and enhances the efficacy of chemotherapy. In line with this dependency, expression of *BCL-XL*, but not *BCL-2* or *MCL1*, directly correlated to the outcome of chemotherapy-treated CRC patients. Our results provide insights to enable the rational use of BH3 mimetics in CRC management, particularly underlining the therapeutic potential of BCL-XL targeting mimetics in both early and late-stage disease.

## Introduction

Intestinal homeostasis is tightly regulated by a balance between proliferation and apoptosis, with intestinal stem cells (ISCs) at the bottom of the crypt driving regeneration [1]. Disruption of this balance is an integral step in colorectal cancer (CRC) initiation and progression. The various pressures associated with oncogenic transformation can activate the apoptosis cascade as an anticancer defense mechanism. Deregulation of the apoptosis machinery to allow uncontrolled proliferation is therefore frequently observed in several tumor types, including CRC [2, 3].

Increasing the apoptotic threshold by modulating the expression or activity of the B-cell lymphoma-2 (BCL-2) family proteins is one such mechanism of evading cell death. The BCL-2 family consists of pro- and antiapoptotic proteins whose interactions dictate whether a cell will undergo mitochondrial outer membrane permeabilization to ultimately activate caspases, the executors of apoptosis [4]. Not surprisingly, tumor cells frequently deregulate BCL-2 family proteins as a mechanism of survival and resistance [5–7]. While BCL-2 is required for apoptosis resistance in several hematological malignancies, amplification of *MCL-1* and *BCL-XL* by chromosomal gain is a frequent alteration especially in solid tumors, making them promising targets for therapy [7]. Inhibiting antiapoptotic proteins with so-called BH3 mimetics holds great promise for anticancer therapy, heralded by the remarkable results obtained with FDA-approved BCL-2 inhibitor Venetoclax (ABT-199), particularly in blood cancers [8].

Although there are promising indications for BCL-2 inhibition in solid tumors [9, 10], BCL-XL is more often found to be upregulated in these tumors and of all tumor types, amplification is most often detected in CRC [7, 11]. Several studies observe an increased expression of BCL-XL in CRC tumors in comparison to normal tissue, where it is crucial for tumor cell survival and resistance [11–15]. In this regard, we have previously shown that colon cancer stem cells (CSCs) present with an increased apoptotic threshold that renders them chemo-refractory, where CSC resistance stems specifically from BCL-XL expression [16]. Interestingly, when analyzing factors that facilitate intestinal transformation, we identified BCL-2 as a critical mediator of this process [17]. ISC-specific knockdown of BCL-2 severely impaired adenoma outgrowth in mice, as did treatment with the specific inhibitor ABT-199 [17]. In the colon, loss of BCL-2 or BCL-XL does not impair intestinal homeostasis while recent data suggest that *MCL1* deletion causes aberrant cell death, thereby prompting Wnt-dependent proliferation that eventually leads to tumor formation, indicating potential detrimental side-effects of targeting this protein [14, 17, 18].

To better assess the role of antiapoptotic proteins in CRC progression, we made use of a panel of genetically-engineered colon organoids that reflect the classical progression pathway of CRC [19–21]. Clonogenic assays with specific BH3 mimetics revealed that ISCs require BCL-2 activity solely during transformation and that this dependence is lost quickly after, while MCL1 inhibition does not affect ISC clonogenicity and BCL-XL is essential for ISC survival throughout CRC progression. Here we show that loss of sensitivity to BCL-2 inhibition is apparent after acquisition of an *APC* mutation, which is mediated by the upregulation of microRNA-17-5p that targets and downregulates BCL-2. Furthermore, we find that BCL-XL inhibition impairs adenoma outgrowth in vivo and augments chemotherapy-induced cell death in tumor-derived organoids. Our results provide the mechanism behind transformation-driven changes in antiapoptotic protein dependence and highlight the therapeutic potential of BCL-XL inhibition in CRC.

## Results

### BCL-2 and BCL-XL are essential for ISC survival during transformation

**Fig. 1:**
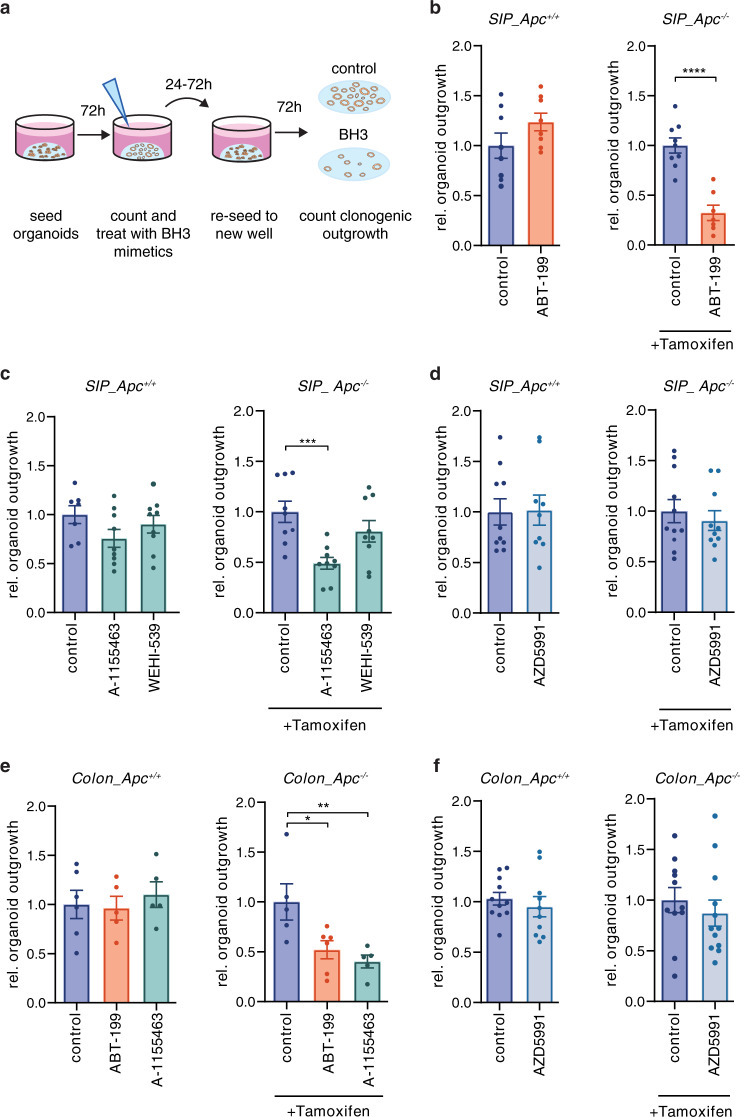
BCL-2 and BCL-XL are essential for ISC survival during transformation. **a** Overview of the organoid clonogenic assay. **b**–**d** Graphs depict relative outgrowth (number of organoid structures relative to the number prior to treatment and reseeding) of proximal small intestine (SIP)-derived organoid structures quantified 3 days after passaging for the indicated genotype, either without treatment or upon treatment with 1 µM **b** ABT-199, **c** A-1155463 and WEHI-539, or **d** AZD5991, at the time of induction. Each dot represents a replicate (minimal *n* = 7 per condition, *n* = 3 independent experiments), error bars indicate s.e.m. ***p < 0.001, ****p < 0.0001, student’s *t*-test. **e**, **f** Graphs depict relative outgrowth of colon-derived organoid structures quantified 3 days after passaging for the indicated genotype, upon treatment with 1 µM **e** ABT-199 and A-1155463 or **f** AZD5991, at the time of induction. Each dot represents a replicate (minimal *n* = 5 per condition, *n* = 2 independent experiments), error bars indicate s.e.m. *p < 0.05, **p < 0.01, ordinary one-way ANOVA.

Previously we have shown BCL-2 to be critical for stem cell survival following loss of *Apc* [17]. We confirmed the importance of BCL-2 for ISC transformation by performing a clonogenic assay with small intestinal organoids from *Lgr5*Cre^ER^*Apc*^fl/fl^ mice. This assay measures survival of the stem cell compartment, which is crucial for the clonogenic potential of the cultures (Fig. 1a). Treatment with BCL-2 specific inhibitor ABT-199 simultaneously with tamoxifen-induced *Apc* loss reduced outgrowth of *Apc*^−/−^ cells, while non-induced organoids remained unaffected by BCL-2 inhibition (Fig. 1b). In the same study, BCL-XL inhibitor WEHI-539 did not affect outgrowth of *Apc*^−/−^ organoids, thus indicating that BCL-XL is nonessential for adenoma survival [17]. However, BH3 profiling has recently shown that WEHI-539 is only weakly efficient in inducing apoptosis in BCL-XL dependent cells while another higher affinity inhibitor, A-1155463, is far more potent [22, 23]. Analyzing both BCL-XL inhibitors confirmed that WEHI-539 did not impair adenoma outgrowth while A-1155463 clearly reduced *Apc*^−/−^ organoid survival during transformation (Fig. 1c). The observed decrease in organoid clonogenicity was not a result of toxicity as wild-type organoids were insensitive to the combination of tamoxifen and BH3 mimetics (Supplementary Fig. 1a). MCL1 deletion has been shown to drastically affect several cell types including the colonic epithelium, however specific MCL1 inhibitors seem to be well tolerated in several preclinical cancer models [18, 24, 25]. To assess the role of MCL1 in ISC transformation, we tested a specific inhibitor, AZD5991, and found that MCL1 targeting did not impair *Apc*^−/−^ organoid outgrowth (Fig. 1d). Taken together, our results provide evidence that BCL-2 and BCL-XL, but not MCL1, are crucial for *Apc*-mutant stem cell survival during transformation.

In contrast to the dependency observed during transformation, we have shown that tumor-derived CSCs are solely BCL-XL-dependent, pointing to a loss of the initial BCL-2 dependence [16]. We confirmed this finding with ABT-199, AZD5991, and A-1155463, where only BCL-XL inhibition induced significant cell death in Wnt-high CSCs, measured by the percentage of cells with active caspase-3 (Supplementary Fig. 1b) [26]. To ensure that the difference in ABT-199 sensitivity between early and late stage disease was not due to a difference between small intestine (SI) and colon-derived cultures, we also tested the sensitivity of *Lgr5*Cre^ER^*Apc*^fl/fl^ colon-derived organoids to BCL-2 and BCL-XL inhibition during transformation and observed strong dependency on both (Fig. 1e), while MCL1 inhibition again had no impact on adenoma outgrowth (Fig. 1f). Our results therefore indicate that while BCL-2 is essential for *Apc*-mutant stem cell survival during transformation, this dependence is reduced at a certain stage during CRC progression.

### BCL-2 dependence is lost immediately after transformation

**Fig. 2:**
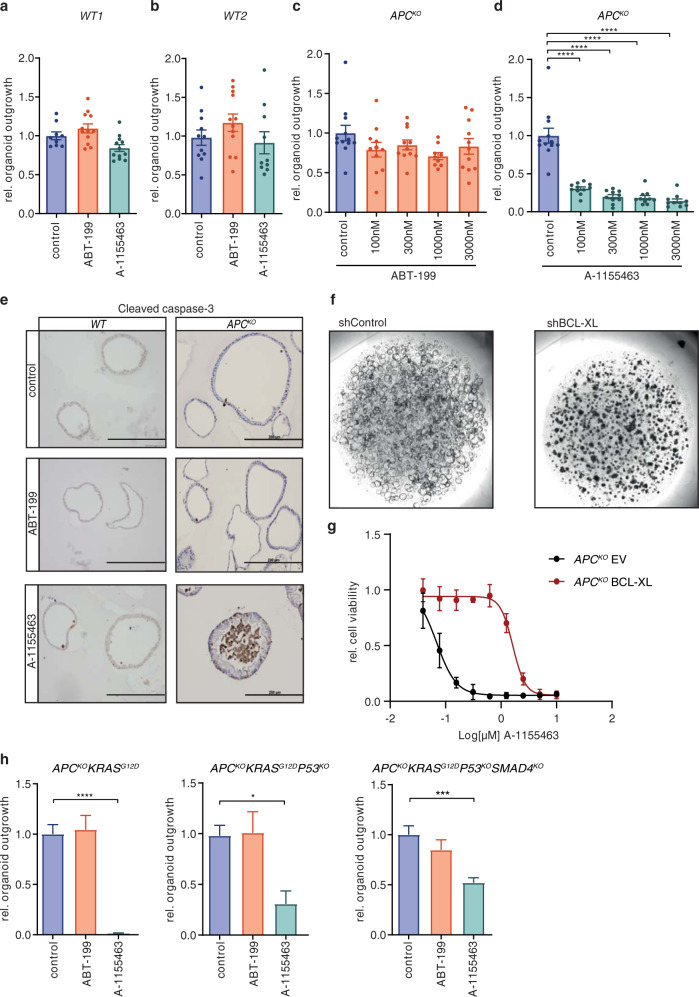
BCL-2 dependence is lost immediately after transformation. **a**, **b** Graphs depict relative outgrowth of human normal colon organoids **a** WT1 and **b** WT2 quantified 3 days after passaging, upon treatment with 1 µM ABT-199 or A-1155463. Each dot represents a replicate (minimal *n* = 9 per condition, *n* = 3 independent experiments), error bars indicate s.e.m. **c**, **d** Graphs depict relative outgrowth of human *APC*^*KO*^ organoids quantified 3 days after passaging, upon treatment with indicated doses of **c** ABT-199 or **d** A-1155463. Each dot represents a replicate (minimal *n* = 9 per condition, *n* = 3 independent experiments), error bars indicate s.e.m. ****p < 0.0001, ordinary one-way ANOVA. **e** Cleaved caspase-3 stained human normal (WT) and *APC*^*KO*^ organoids after 24 h treatment with 300 nM ABT-199 or A-1155463. Scale bars, 200 µm. **f** Images represent the outgrowth of *APC*^*KO*^ organoids after transduction with either control or BCL-XL targeting shRNA. **g** Dose-response curve of control vector (black) or BCL-XL overexpressing (red) *APC*^*KO*^ organoids treated with a titration of A-1155463. Viability data measured by cell titer blue was blank corrected and normalized to untreated control. Data were represented as mean ± SD (*n* = 2 independent experiments). **h** Relative outgrowth of human mutant organoids of indicated genotypes quantified 3 days after passaging, upon treatment with 3 µM of ABT-199 or A-1155463 (*n* = 3 independent experiments). Error bars indicate s.e.m. *p < 0.05, ***p < 0.001, ****p < 0.0001, ordinary one-way ANOVA.

The most frequently mutated genes in the classical CRC progression pathway, namely *APC, KRAS, P53*, and *SMAD4*, were previously altered in human colon-derived organoids using CRISPR-Cas9-mediated genome editing [20]. We employed this panel of organoids to assess changes in antiapoptotic protein dependence of ISCs. Clonogenic outgrowth of normal colon organoids was unaffected by treatment with either ABT-199 or A-1155463 (Fig. 2a, b) while in *APC* knock-out (*APC*^*KO*^) organoids, ABT-199 treatment had no effect (Fig. 2c) and A-1155463 treatment strongly impaired organoid clonogenicity (Fig. 2d). To corroborate this finding, we analyzed activation of apoptosis by cleavage of caspase-3 and found clear induction only in *APC*^*KO*^ organoids treated with A-1155463, indicating that apoptosis is induced in these organoids upon BCL-XL, but not BCL-2, inhibition (Fig. 2e). Similarly, mouse *Apc*^−/−^ adenomas were also insensitive to ABT-199 treatment while A-1155463 clearly impaired adenoma outgrowth, indicating that this is not a difference between mouse and human-derived cultures (Supplementary Fig. 2a, b). To exclude that A-1155463 induced apoptosis could arise from off-target effects, shRNA knockdown of *BCL-XL* was performed. Strikingly, *BCL-XL* knockdown severely impaired survival of *APC*^*KO*^ organoids, while knockdown with a control shRNA had no effect (Fig. 2f and Supplementary Fig. 2c). Reciprocally, BCL-XL overexpression induced significant resistance to A-1155463 in *APC*^*KO*^ organoids (Fig. 2g and Supplementary Fig. 2d), thus confirming that impaired clonogenicity upon A-1155463 treatment was a direct result of BCL-XL inhibition. In addition, we tested MCL1 dependency with AZD5991, which had no effect on the clonogenicity of both wild-type and *APC*^*KO*^ organoids (Supplementary Fig. 2e, f). This pattern of sensitivity to BCL-XL inhibition and absence of BCL-2 and MCL1 dependency in transformed ISCs was maintained throughout the progression panel of organoids with increasing mutation loads (Fig. 2h and Supplementary Fig. 2g). Altogether these results indicate that BCL-2 exerts its pro-survival functions during transformation alone while BCL-XL is required for apoptosis evasion throughout the adenoma-to-carcinoma sequence. Intriguingly, triple (*APC*^*KO*^*KRAS*^*G12D*^*P53*^*KO*^) and quadruple (*APC*^*KO*^*KRAS*^*G12D*^*P53*^*KO*^*SMAD4*^*KO*^) mutant organoids did show slightly increased resistance to A-1155463, which is consistent with the role of P53 in apoptosis induction [27]. In agreement, shRNA-mediated knockdown of *Smad4* in mouse *Apc*^*−/−*^*Kras*^*+/G12D*^ organoids did not induce increased resistance to A-1155463, while quadruple mutant organoids were again more resistant (Supplementary Fig. 2h). This P53 mutation induced resistance to A-1155463 is likely due to the observed downregulation of its known proapoptotic targets such as *PUMA* and *BAX* [27, 28] (Supplementary Fig. 7e, f).

### BCL-XL is crucial for stem cell survival in patient-derived adenoma and tumor organoids

**Fig. 3:**
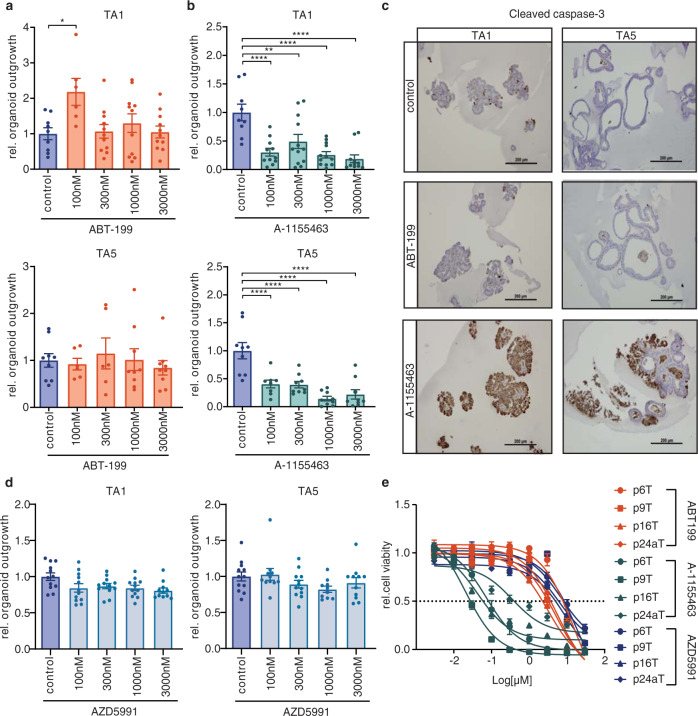
BCL-XL is crucial for stem cell survival in patient-derived adenoma and tumor organoids. **a**, **b** Graphs depict relative outgrowth of human tubular adenomas TA1 and TA5 quantified 3 days after passaging, upon treatment with indicated doses of **a** ABT-199 or **b** A-1155463. Each dot represents a replicate (minimal *n* = 6 per condition, *n* = 3 independent experiments), error bars indicate s.e.m. *p < 0.05, **p < 0.01, ****p < 0.0001, ordinary one-way ANOVA. **c** Cleaved caspase-3 stained human TAs after 24 h treatment with 300 nM ABT-199 or A-1155463. Scale bars, 250 µm. **d** Graphs depict relative outgrowth of human tubular adenomas TA1 and TA5 quantified 3 days after passaging, upon treatment with indicated doses of AZD5991. Each dot represents a replicate (minimal *n* = 6 per condition, *n* = 3 independent experiments), error bars indicate s.e.m. **e** Relative viability of patient-derived CRC organoids upon treatment with a titration of ABT-199 (orange), A-1155463 (green), or AZD5991 (blue) (*n* = 3 independent experiments). Error bars indicate s.e.m.

To ascertain the clinical relevance of the above findings, we tested the response of organoids derived from familial adenomatous polyposis (FAP) and CRC patients to antiapoptotic protein inhibition. Tubular adenomas (TA) derived from FAP patients represent precursor lesions that have an increased likelihood of developing into carcinomas and are normally the result of loss or mutation of the wild-type *APC* allele [19]. All four tested TA cultures were insensitive to increasing doses of BCL-2 inhibition with ABT-199 and treatment did not induce cleavage of caspase-3, similar to the *APC*^*KO*^ human organoids (Fig. 3a, c and Supplementary Fig. 3a, c). Conversely, BCL-XL inhibition strongly impaired adenoma outgrowth and A-1155463 treated TAs showed elevated expression of cleaved caspase-3 (Fig. 3b, c and Supplementary Fig. 3b, c). Also in this setting, MCL1 inhibition did not impair adenoma clonogenicity, in agreement with the minimal impact of AZD5991 on *APC*^*KO*^ organoids (Fig. 3d).

Tumor organoids established from patient-derived CRC biopsies have been shown to closely reflect several characteristics of the original tumor [21]. We tested four of these tumor organoids (p6T, p9T, p16T, and p24aT), all of which were previously confirmed to have alterations in *APC, KRAS*, and *P53*, with p16T also carrying a *SMAD4* mutation [21]. As expected, all four tumor organoids were more sensitive to BCL-XL inhibition as the IC50s for A-1155463 were much lower than for ABT-199 and AZD5991 (Fig. 3e), confirming that classical CRC precursor and carcinoma lesions depend on BCL-XL for survival while BCL-2 and MCL1 remain nonessential in this context.

### BCL-XL inhibition impairs adenoma outgrowth in vivo

**Fig. 4:**
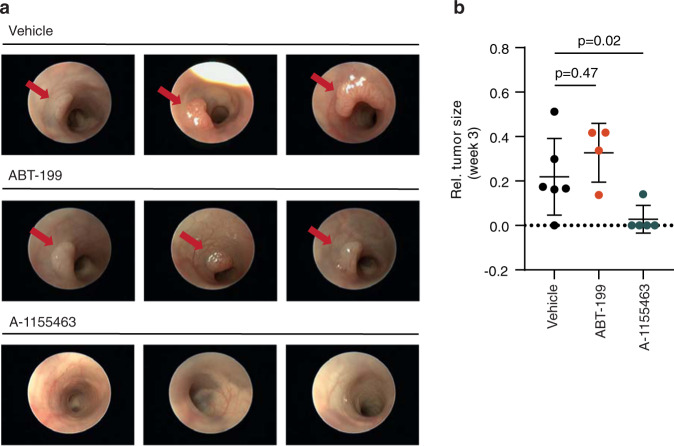
BCL-XL inhibition impairs adenoma outgrowth in vivo. **a** Representative images from a colonoscopy of *Villin*Cre^ER^*Apc*^fl/fl^ mice, three weeks after being induced with a single injection of 4-hydroxytamoxifen into the colonic submucosa. ABT-199 and A-1155463 treated mice received 100 mg/kg of drug for 2 days prior to induction followed by treatment every other day until day 28. Red arrows indicate tumors. **b** Quantification of colonic tumor size (normalized to the size of the lumen) of mice described in (**a**), *n* = 6 for vehicle, *n* = 4 for ABT-199, and *n* = 5 for A-1155463, one-way Mann–Whitney *U*-test, *p* = 0.47 (vehicle vs ABT-199) and *P* = 0.02 (vehicle vs A-1155463). Data represents mean ± SD.

Our results suggest that the window for BCL-2 inhibition as a therapeutic strategy for CRC is limited and that MCL1 inhibition on its own is unlikely to be effective. However, BCL-XL inhibition could prevent tumor outgrowth at early stages. To test this in vivo, we injected 4- hydroxytamoxifen into the colonic submucosa of *Villin*Cre^ER^*Apc*^fl/fl^ mice to induce local recombination and orally administered either ABT-199 or A-1155463, once every two days for the duration of the experiment. Small tumors were visible upon colonoscopy in vehicle and ABT-199 treated groups after 1 and 2 weeks, while none were detected in the A-1155463 treated mice (Supplementary Fig. 4a, b). After 3 weeks, all of the ABT-199 treated mice had discernable adenomas while only one of five A-1155463 treated mice presented with a small adenoma (Fig. 4a, b), thus confirming that BCL-XL inhibition effectively attenuates adenoma outgrowth in vivo. Intriguingly, BCL-XL inhibition was not effective on preexisting adenomas in this model (Supplementary Fig. 4c, d), which is likely due to the higher dose of A-1155463 needed to achieve the same level of apoptosis in fully transformed organoids (Supplementary Fig. 2b) in comparison to transforming organoids (Supplementary Fig. 4e).

### BCL-2 expression is decreased upon transformation

**Fig. 5:**
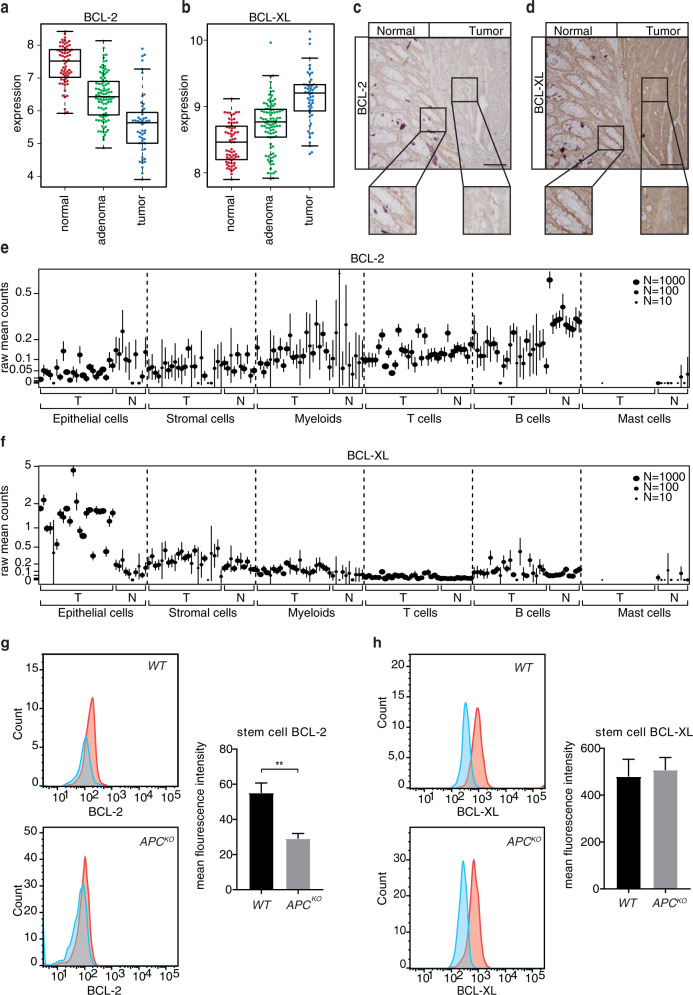
BCL-2 expression is decreased upon transformation. **a**, **b** mRNA expression of **a** *BCL-2* and **b** *BCL-XL* in a compiled dataset of colon normal, adenoma and tumor samples. **c**, **d** Immunohistochemical analysis of **c** BCL-2 and d BCL-XL expression in normal and adjacent tumor tissue. Scale bars, 200 µm. **e**, **f** Gene expression (raw mean counts) of **e** *BCL-2* and **f** *BCL-XL* in a single cell RNA-Seq dataset of normal and tumor samples, separated by cell type. Data were represented as mean ± SE and node size indicates the number of cells per sample. Samples in different cell types are shown in the same order. *p* < 0.005 for the fold change in *BCL-2* and *BCL-XL* expression in normal vs tumor epithelial cells. **g**, **h** Intracellular FACs staining of **g** BCL-2 and **h** BCL-XL in the PTK7 high stem cell fraction of normal and *APC*^*KO*^ human organoids. (*n* = 3 independent experiments). Error bars indicate s.e.m. **p < 0.01, student’s *t*-test.

In a study that assessed determinants of BH3 mimetic efficacy, sensitivity to BCL-2 inhibition could be predicted by expression levels of the protein, where BCL-2 expressing cells respond better to ABT-199 than non-expressers [29]. Having established BCL-2 inhibition to be ineffective after ISC transformation, we therefore determined whether this relates to its expression pattern during CRC progression by analyzing four publicly-available expression datasets. This revealed a significant decrease in *BCL-2* expression in adenomas compared to healthy tissue and a further decrease in CRC tumors, which was also confirmed in the TCGA COREAD cohort (Fig. 5a and Supplementary Fig. 5a). Conversely, *BCL-XL* expression increased upon progression from normal to adenoma and furthermore in the carcinoma stage (Fig. 5b and Supplementary Fig. 5b). At the protein level, BCL-2 was detected in normal crypts, however its expression was nearly absent in adjacent tumor tissue, while BCL-XL showed strong positive staining in both normal and tumor tissue (Fig. 5c, d). Independent confirmation with a single-cell RNA sequencing dataset of normal and CRC tumor samples [30] revealed a significant decrease in *BCL-2* and concomitant increase in *BCL-XL*, specifically in the epithelial tumor compartment (Fig. 5e, f). Based on these observations, we next ascertained whether the observed decrease in BCL-2 expression occurs already upon acquisition of an *APC* mutation, specifically in the stem cell compartment where BCL-2 is specifically expressed [17]. To do so, we made use of a validated colon stem cell marker, PTK7, and confirmed its function as a stem cell marker for *APC*^*KO*^ human organoids as well [31] (Supplementary Fig. 5c, d). Analysis of BCL-2 expression in PTK7-high stem cells of normal and *APC*^*KO*^ human organoids confirmed a decrease in stem cell BCL-2 expression upon loss of *APC* (Fig. 5g and Supplementary Fig. 5e, g). A similar analysis of BCL-XL expression showed no change in expression between normal and *APC*^*KO*^ stem cells (Fig. 5h and Supplementary Fig. 5f, g). Our data thus indicates that transformation results in a decrease in BCL-2 expression, which explains the observed lack of impact of BCL-2 inhibition in *APC*^*KO*^ and TA cultures.

### MiR-17-5p regulates BCL-2 expression in APC-mutant organoids

**Fig. 6:**
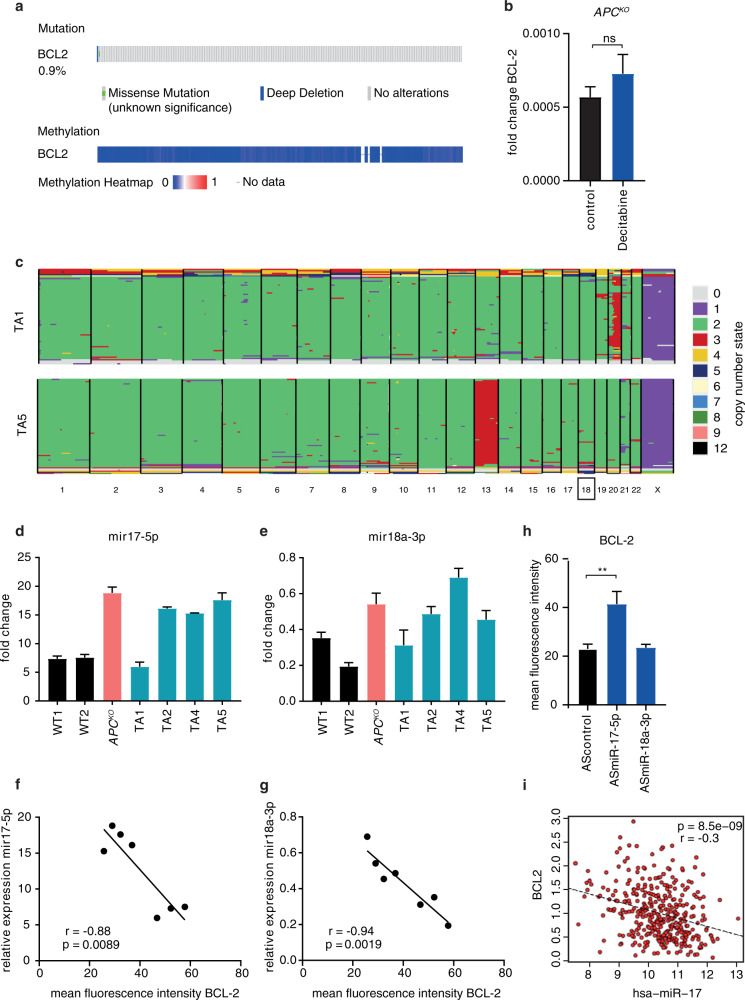
MiR-17-5p regulates BCL-2 expression in *APC*-mutant organoids. **a** *BCL-2* mutation and methylation status in CRC tumors of the TCGA COAD dataset, analyzed on cBioPortal. **b** mRNA expression of *BCL-2* in decitabine treated *APC*^*KO*^ organoids (*n* = 2 independent experiments). Error bars indicate s.e.m. **c** Single cell karyotype-seq showing the ploidy in individual cells of TA1 and TA5. Graphs show individual cells (horizontal lines) and colors indicate copy number state for a given chromosome (columns). **d**, **e** qRT-PCR analysis of **d** miR-17-5p and **e** miR-18a-3p expression in human normal, *APC*^*KO*^ and TA cultures, normalized to U6 snRNA expression. Data represent mean ± s.e.m. **f**, **g** Correlation of **f** miR-17-5p and **g** mir-18a-3p expression data from (**d**) and (**e**), respectively to BCL-2 protein levels measured by intracellular FACS. *P* values are determined by two-tailed Pearson’s correlation. **h** Intracellular FACS staining of BCL-2 protein levels in human *APC*^*KO*^ organoids transduced with control, miR-17-5p, or miR-18a-3p anti-sense(AS) microRNA inhibitors (*n* = 3 independent experiments). Error bars indicate s.e.m. **p < 0.01, student’s *t*-test. **i** Correlation of miR-17 and *BCL-2* expression in the TCGA COAD dataset.

Next, we assessed the mechanism by which BCL-2 expression levels decrease upon transformation. We first evaluated *BCL-2* mutation status by analyzing the TCGA COAD dataset on cBioPortal, where most tumors did not present with any alterations in the gene (Fig. 6a). Evaluation of promoter methylation in the same dataset showed an absence of methylation in the *BCL-2* promoter region (Fig. 6a), further confirmed by treatment of *APC*^*KO*^ organoids with the demethylating agent decitabine, which did not affect *BCL-2* mRNA levels (Fig. 6b). The *BCL-2* gene is located on 18q and could therefore be lost as a consequence of loss of heterozygosity (LOH) of this chromosomal region, which frequently occurs in CRC, albeit at a later stage of the disease [32–34]. However, karyograms of *APC*^*KO*^ human organoids did not show loss of 18q [20]. Similarly, single-cell karyotype sequencing to quantify copy number alterations revealed no 18q LOH in any of the TA cultures, thereby excluding LOH as the underlying mechanism of decreased *BCL-2* expression in adenomas (Fig. 6c and Supplementary Fig. 6a).

Several microRNAs show aberrant expression in colon adenoma and carcinoma samples in comparison to normal tissue [35]. We examined the most commonly upregulated microRNAs in early adenomas and found several of them to have binding sites on the *BCL-2* 3′UTR, as predicted by miRmap (Supplementary Table 1) [35, 36]. Of these, the miR-17-92 family was of particular interest as it is a direct target of β-catenin, upregulated upon mutation in the *APC* gene [37]. Several members of this microRNA family have high miRmap scores for binding in the 3′UTR of *BCL-2* (Supplementary Table 1) [36]. Of note, miR-17 and miR-18a have previously been shown to target *BCL-2* in a luciferase reporter assay and one or more binding sites for miR-17-5p and miR-18a-3p are present on the *BCL-2* 3′UTR [38] (Supplementary Fig. 6b, c). We therefore examined the expression of these microRNAs in our human normal, *APC*^*KO*^ and TA organoids and found that both miR-17-5p and miR-18a-3p were increased in *APC*^*KO*^ organoids and in the majority of TA cultures (Fig. 6d, e). A significant negative correlation between the protein levels of BCL-2 and the expression of both miR-17-5p and miR-18a-3p was also observed in these organoids (Fig. 6f, g). To assess if these microRNAs mediate the downregulation of BCL-2, we transduced *APC*^*KO*^ organoids with lentiviral microRNA inhibitors that target either mir-17-5p or mir-18a-3p. Protein expression of BCL-2 was found to be significantly upregulated upon mir-17-5p inhibition, but not upon mir-18a-3p inhibition (Fig. 6h and Supplementary Fig. 7a). We confirmed the efficacy of mir-17-5p inhibition with the observed upregulation of BIM, a known target of mir-17 (Supplementary Fig. 7b) [39]. MYC, another mir-17 target [40], was not altered at the mRNA level and was lowly expressed in these cells (Supplementary Fig. 7c, d). Importantly, BCL-XL, which is not a miR-17 target, remained unaffected (Supplementary Fig. 7b). Furthermore, we observed a significant negative correlation between miR-17 and *BCL-2* levels in the TCGA dataset of micro-satellite stable (MSS) CRC tumors (Fig. 6i). Our results thus confirm that *APC*-driven transformation results in upregulation of mir-17-5p, which mediates repression of BCL-2.

Altogether, our data explain why ISC transformation results in a rapid decrease in BCL-2 expression and as a consequence, a decrease in BCL-2 dependency. However, to elucidate why transformation results in an increased sensitivity to A-1155463 while normal ISCs are resistant, we profiled the expression of key members of the BCL-2 family and assessed if transformation-driven changes to the apoptotic threshold could explain this shift in BCL-XL dependency [41, 42]. Surprisingly, mRNA and protein levels of several proapoptotic proteins were decreased upon transformation. Nevertheless, we also observed a dramatic decrease in MCL1 levels, which, together with the above described repression of BCL-2, results in evident reliance on BCL-XL for survival, thereby making it a key vulnerability in transformed ISCs (Supplementary Fig. 7e, f).

### BCL-XL expression is predictive for chemotherapy response

**Fig. 7:**
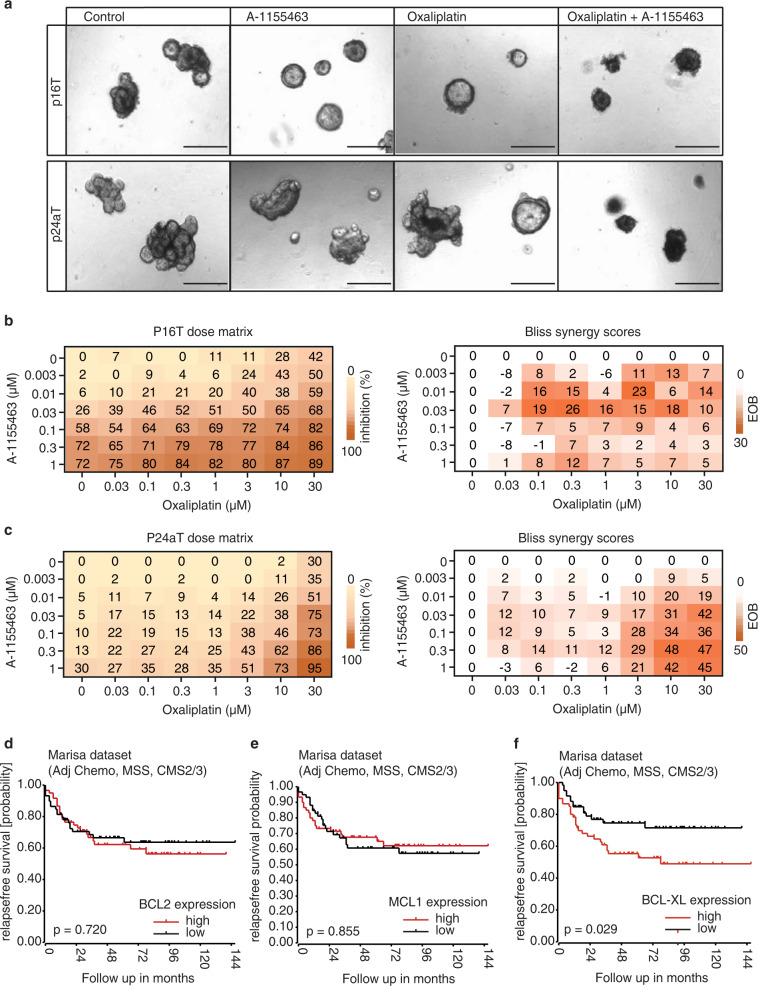
BCL-XL expression is predictive for chemotherapy response. **a** Phase-contrast images of p16T and p24aT human CRC organoids treated for 72 h with 10 µM oxaliplatin alone and in combination with 30 nM A-1155463. Scale bars, 250 µm. **b**, **c** 6 × 7 dose matrices of **b** p16T and **c** p24aT human CRC organoids treated with oxaliplatin in combination with A-1155463 for 5 days. Percentage inhibition was calculated from viability data measured by cell titer blue, after normalizing to control. Data were the average of two independent experiments. Bliss synergy scores were calculated for each dose combination and positive scores indicate synergistic effects. **d**–**f** Kaplan–Meier analysis of the relapse free survival probability of micro-satellite stable CM2 and CM3 patients in the Marisa dataset who received adjuvant chemotherapy (*n* = 119), based on a cut-off of median expression of **d** *BCL-2*, **e** *MCL-1*, or **f** *BCL-XL*. *P* values are based on the log-rank test. Adj Chemo patients who received adjuvant chemotherapy, MSS microsatellite stable, CMS2/3 consensus molecular subtype 2/3.

Our data show that BCL-XL plays a critical role in CRC survival, yet the in vivo use of A-1155463 was ineffective in impairing preexisting adenoma growth (Supplementary Fig. 4c, d). We therefore determined if BCL-XL inhibition could enhance the efficacy of chemotherapy on patient-derived tumor organoids. Matrix titration of A-1155463 and Oxaliplatin revealed that the efficacy of the two compounds is strongly synergistic (Fig. 7a–c and Supplementary Fig. 8a–c). Intriguingly, studies have shown that the proximity of tumor cell mitochondria to the apoptotic threshold is also predictive of chemotherapy response in patients [43]. We therefore analyzed if the changes we detected in antiapoptotic protein reliance have clinical relevance in terms of outcome in chemotherapy-treated nonmetastatic CRC patients. To exclude variation in chemotherapy response due to CRC subtype differences, we made use of the Marisa dataset and selected the microsatellite stable epithelial subtypes CMS2 and CMS3 (*n* = 119), which almost invariably carry an *APC* mutation and are therefore representative of the observed *APC*-driven decrease in BCL-2 expression. Kaplan–Meier analysis for relapse-free survival was performed with a cut-off based on median expression of *BCL-2, MCL1*, or *BCL-XL*. While *BCL-2* and *MCL1* expression did not differentiate survival probability (Fig. 7d, e), patients with low *BCL-XL* expression and therefore a lower apoptotic threshold displayed a more favorable response to adjuvant chemotherapy in comparison to the high *BCL-XL* expressors (Fig. 7f), thus suggesting that the extent of BCL-XL-driven protection from mitochondrial apoptosis is predictive for response to chemotherapy.

## Discussion

The development of BH3 mimetics has greatly facilitated our understanding of the role of antiapoptotic proteins in normal and disease settings. In particular, their specificity for individual BCL-2 family members allows for dissecting the antiapoptotic vulnerabilities of cancer cells and thereby define enhanced therapeutic strategies. By employing these powerful tools, we show that while BCL-2 is only essential during ISC transformation, BCL-XL is critical for ISC survival throughout CRC progression. Previous work has shown that transforming B lymphoid cells present with an increased sensitivity to MCL1 loss [44]. However, we find that AZD5991 treatment does not impair ISC transformation in our models, even though MCL1 deletion was shown to severely disrupt intestinal homeostasis [18]. Earlier observations indicate that MCL1 deletion is not directly comparable to its pharmacological inhibition, which is often well tolerated at therapeutically effective doses [18, 24]. We and others have shown that while most solid tumors including CRC do not respond to MCL1 inhibition, combined inhibition of MCL1 and BCL-XL is very potent, suggesting that high levels of BCL-XL deter the efficacy of MCL1 inhibition alone [24, 25, 29, 45]. Overall, our data indicates that BCL-2 and MCL1 expression is rapidly lost upon transformation and that this results in a shift towards increased BCL-XL dependency. Our data further solidifies BCL-XL as a key factor for apoptosis resistance in CRC and moreover, emphasizes its importance in early stages of the disease. Patient-derived organoids have been used as a model to predict patient response to chemotherapy [46] and our results highlight the therapeutic potential for BCL-XL inhibition in CRC as A-1155463 treatment effectively impairs viability of organoids derived from FAP and CRC patients.

The use of BCL-XL inhibitors in the clinic is hampered by their toxicity to platelets, whose lifespan is determined by BCL-XL expression [47]. However, this could be circumvented with altered dosing strategies, particularly by administering lower doses in combination with other chemotherapeutics. Another possibility for circumventing platelet toxicity and still achieving CRC tumor killing would be to use BCL-XL inhibitors with reduced oral bioavailability, which could particularly work in the context of intestinal tumors. We therefore used A-1155463 instead of its more orally bioavailable counterpart A-1331852 in our in vivo experiments, which showed considerable efficacy in reducing adenoma outgrowth with no observed toxicity, indicating potential for less orally bioavailable BCL-XL inhibitors in CRC therapy. However, oral A-1155463 treatment on its own did not affect the growth of established adenomas in mice, which could be due to inadequate dosing of A-1155463 as transformed cells are not as sensitive to its inhibition as transforming cells. In this case, using higher doses of less orally bioavailable BCL-XL inhibitors would offer potential to improve treatment efficacy but more so, identifying kinase targets whose inhibition could synergize with BCL-XL targeting could provide novel strategies to target CRC tumors. Here we show that BCL-XL inhibition enhances oxaliplatin-induced apoptosis, while earlier studies also observed synergy with inhibitors of kinases such as MEK [48, 49].

Previously, we have shown BCL-2 inhibition with ABT-199 to effectively reduce adenoma burden in vivo [17] while in our study this effect is no longer apparent. However, in our in vivo experiment tumor formation was localized to the colon rather than the SI as was the case in the previous study and so we reason that the orally bioavailable ABT-199 is likely absorbed through the SI and fails to reach the tumor in the colon. The limited window of ABT-199 efficacy that we observe in our organoid progression panel is supported by the decrease in BCL-2 expression detected upon transformation. In the context of tumorigenesis, such a decrease in antiapoptotic protein expression is counterintuitive. With the aim to resolve this, we identified miR-17-5p as a BCL-2 repressor that is significantly upregulated upon acquisition of an *APC* mutation. In CRC, miR-17-5p expression increases as the disease progresses and is suggested to be regulated by transcription factors such as MYC and β-catenin [37, 50, 51]. MiR-17-5p has therefore been implicated to have an oncogenic role in the disease and higher expression is associated with a lower overall survival [52–54]. We propose that the observed decrease of BCL-2 is an inadvertent consequence of transformation-driven miR-17-5p upregulation, whose oncogenic potential might simply outweigh the antiapoptotic contribution of BCL-2.

Dynamic regulation of apoptosis has been observed during development as tissues progress from a young, proliferative state to a more aged and differentiated state, where MYC acts as a link between cell growth and death [55]. MYC high cells found in younger tissues pay the price for their proliferation with increased apoptotic priming, making them more sensitive to death-inducing stimuli. Such an effect is also observed in tumor cells, particularly driven by oncogenic mutations and regulated by MYC levels [56, 57]. Cancer cells are therefore in a precarious position where they require their antiapoptotic block to hold in order to ensure their survival. This provides a potential therapeutic strategy and better identification of these transformation-induced vulnerabilities could provide valuable targets for therapy. Here we report transformation-driven changes in antiapoptotic protein expression and dependence during CRC initiation and progression. Our findings could guide the rational use of BH3 mimetics as anticancer agents, whose clinical application can be aided by evaluating antiapoptotic dependencies in varying tumor landscapes.

## Materials and methods

### Mouse organoid isolation and culture

Intestinal crypts were derived from the SI and colon of a wild-type and *Lgr5*Cre^ER^*Apc*^fl/fl^ mouse. Crypts were isolated and cultured as described previously [58]. Briefly, the intestine was isolated and separated into SI and colon pieces. After flushing with PBS, the intestines were opened longitudinally and villi were scraped off the SI. Both sections were then cut into small pieces of ~0.5 cm and washed thoroughly with cold PBS supplemented with antibiotics (hereon referred to as PBS + antibiotics). These pieces were then incubated with 2 mM (SI) and 25 mM (colon) EDTA (Thermo Fischer Scientific, Landsmeer, The Netherlands) in PBS at 4 °C on a roller for 30 min. Crypts were isolated by washing the pieces with PBS + antibiotics and vigorously shaking to release crypts into the supernatant, which was collected and filtered through a 70 µm strainer (BD Biosciences, Breda, The Netherlands). Crypts were then spun down and plated in growth factor-reduced Matrigel (Corning, Amsterdam, The Netherlands) in advanced DMEM/F12 medium supplemented with N2 and B27 supplement (Thermo Fischer Scientific), 2 mM GlutaMAX-I (Thermo Fischer Scientific), 10 mM Hepes (Thermo Fischer Scientific), 1 mM *N*-acety-l-cysteine (Sigma-Aldrich, Zwijndrecht, The Netherlands), 1X antimycotic/antibiotic (Thermo Fischer Scientific), mouse EGF 50 ng/ml (Tebu-BIO, Heerhugowaard, The Netherlands), 20% R-spondin (conditioned medium), and 10% Noggin (conditioned medium). Mouse *Apc*^*−/*−^*Kras*^*+/G12D*^ organoids with shRNA knockdown of *Smad4* and *p53* were generated and selected as previously described [28]. All organoid cultures were confirmed to be mycoplasma negative on a monthly basis.

### Human organoid isolation and culture

Normal colon samples (WT1 and WT2; WT1 is the normal organoid source of the CRISPR-Cas9 generated mutant organoids) were obtained from a piece of normal mucosa in the resection specimens of two CRC patients, at a distance of at least 10 cm from the cancerous tissue. Normal organoids were isolated as previously described [59]. In short, the submucosal layer was removed and the mucosal piece was washed with PBS + antibiotics. The tissue was then cut into small pieces using a scalpel and washed thoroughly with PBS + antibiotics. The cut pieces were incubated in 8 mM EDTA in PBS at 4 °C on a roller for 1 h. The EDTA solution was replaced with PBS + antibiotics and crypts were released by vigorous shaking. The supernatant containing crypts was transferred to a new tube and this step was repeated three times. The crypts were then spun down, pooled, and seeded in Matrigel. Normal colon organoids were maintained in Advanced DMEM/F12 supplemented with N2 and B27 supplement, antimycotic/antibiotic, gentamicin (Thermo Fischer Scientific), 2 mM GlutaMAX-1, 10 mM HEPES, 1 mM *N*-acety-l-cysteine, 10 nM [Leu15]-gastrin I (Sigma-Aldrich), 10 mM nicotinamide (Sigma-Aldrich), 500 nM A83-01 (Tocris, Abingdon, UK), 3 µM SB202190 (Sigma-Aldrich), 50% WNT3A conditioned medium, 50 ng/ml human EGF (Peprotech, London, UK), 20% RSPO1 conditioned medium, 10% Noggin conditioned medium, 10 nM PGE2 (Santa Cruz Biotechnology, Heidelberg, Germany), and 10 µM ROCK inhibitor (Sigma-Aldrich). Normal organoids were dissociated every 7–10 days and medium was refreshed every 2/3 days.

The CRIPSR-Cas9 derived mutant panel of organoids were generated as previously described [20]. Normal human colon organoids were transfected with plasmids expressing Cas9 endonuclease and sgRNAs targeting *APC, KRAS, P53*, or *SMAD4*. In the case of *KRAS*, homologous recombination to introduce the G12D mutation was achieved by co-transfection with a targeted oligonucleotide containing the oncogenic mutation. Following appropriate selection, individual organoids were picked and expanded to obtain clonal populations. The sgRNA sequences have been previously described [20] and genotyping was performed to confirm mutations. Mutant organoids were cultured in normal colon organoid medium as described above, excluding the WNT3A conditioned medium.

Human tubular adenoma cultures TA1, TA2, TA4, and TA5 were isolated and cultured as previously described [19]. Tissues were obtained from resections of FAP patients and polyps were cut into small pieces, washed, and plated in Matrigel. Following mechanical dissociation, viable organoids were propagated in Advanced DMEM/F12 containing N2 and B27 supplement, gentamycin, 2 mM l-glutamine (Lonza, Breda, The Netherlands), 0.15% d-glucose (Sigma-Aldrich), 100 µM β-mercaptoethanol (Sigma-Aldrich), trace elements B and C (Thermo Fischer Scientific), 5 mM HEPES, 2 µg/ml heparin (Sigma-Aldrich), 10 µg/ml insulin(Sigma-Aldrich), 20 ng/ml human EGF, and 10 µM SB202190.

Patient-derived tumor organoids were obtained from the Clevers organoid biobank and established as previously described [21]. P6T was cultured in adenoma medium and P9T, P16T, and P24aT were cultured in normal colon organoid medium, excluding the WNT3A conditioned medium. All organoid cultures were tested for mycoplasma on a monthly basis and were confirmed to be mycoplasma negative.

### Organoid transduction

For the shRNA knockdown experiment, an equal number of *APC*^*KO*^ organoids were transduced with lentiviral shRNA constructs against either control (SHC002, MISSION shRNA, Merck,) or BCL-XL (TRCN0000033500, Mission shRNA, Merck) by spin transduction. Briefly, organoids were collected and trypsinized using TrypLE Express (Thermo Fischer Scientific) for 3 min at 37 °C. Following dissociation, cells were washed, counted, and seeded into 48-well plates in organoid medium (see above) containing 10 µM ROCK inhibitor (Sigma-Aldrich), 8 µg/ml polybrene (Sigma-Aldrich), and 50 µL concentrated virus (AMICON Ultra-15 100k filters, Merck, Schiphol-Rijk, The Netherlands). Plates were spun down at 32 °C for 1 h at 1800 rpm. After ON incubation, cells were collected by washing with PBS and spun down and either processed for RNA extraction or seeded into Matrigel (Corning) for measuring outgrowth. *APC*^*KO*^ organoids were transduced with lentiviral pHEFTIR-EV (empty vector) or pHEFTIR-BCL-XL (overexpressor) constructs [16] and selected by sorting for the RFP positive population. *APC*^*KO*^ organoids were transduced with lentiviral microRNA inhibitors (Merck) targeting hsa-miR-17-5p (HLTUD0264), hsa-miR18a-3p (HLTUD0292), and a negative control cel-mir-243-3p (HLTUD002C). Transduced cells were selected with 4 µg/ml puromycin (InvivoGen, Toulouse, France) for 7 days.

### Colon cancer spheroid culture

The colon cancer spheroid culture Co01 expressing the TCF/LEF reporter TOP-GFP was generated as previously described [26]. The spheroid culture was maintained in ultralow adherent flasks (Corning) and cultured in advanced DMEM/F12 supplemented with N2 supplement, 2 mM l-glutamine, 0.15% d-glucose, 100 µM β-mercaptoethanol, trace elements B and C, 5 mM HEPES, 2 µg/ml heparin, 10 µg/ml insulin, 50 ng/ml epidermal growth factor, and 10 ng/ml basic fibroblast growth factor (Tebu-BIO). Cultures were confirmed to be mycoplasma negative on a monthly basis.

### Inhibitors

ABT-199 and WEHI-539 were purchased from Selleck Chemicals (Breda, The Netherlands), A-1155463 and AZD5991 from Chemietek (Indianapolis, USA). 5-Aza-2′-deoxycytidine (Decitabine) and Oxaliplatin were purchased from Sigma Aldrich. All compounds were dissolved in DMSO to a stock of 20 mM, except Oxaliplatin, which was dissolved in water to a stock of 12.5 mM.

### Clonogenic assay

The clonogenic assay on *Lgr5*Cre^ER^*Apc*^fl/fl^ derived SI and colon organoid cultures was performed as previously described [58]. Briefly, organoids were plated in 48-well plates at a density of 25–50 crypt structures per well. After 3 days, the organoids were counted and treated with 1 µM (Z)−4-hydroxytamoxifen (24 h) to induce *Apc* loss and simultaneously treated with 1 µM BH3 mimetics for 72 h. To assess the clonogenic potential of the organoids, each well was passaged and the resulting organoid outgrowth was quantified after 3 days and normalized to the pretreatment count per well and compared to untreated controls.

The clonogenic assay on the human organoids was performed by seeding dissociated organoids in 48-well plates, following which they were treated after 3 days with the indicated dose of BH3 mimetics for 24 h. Each well was then passaged to a new well and the clonogenic capacity of organoids was assessed by microscope after 3 days and compared to untreated controls. All clonogenic assays were performed as two or three independent experiments and include at least three technical replicates per run.

### Flow cytometry

Co01 expressing TOP-GFP was dissociated with Trypsin and stained for activated caspase-3 after 24 h of treatment with 1 µM ABT-199 or A-1155463. Staining was performed using RED-DEVD-FMK (CaspaTag caspase 3,7 in situ assay kit, sulforhodamine, Merck) as per the manufacturer’s instructions (*n* = 2 independent experiments). Organoids were isolated for FACS staining using cell recovery solution (BD Biosciences) at 4 °C for 30 min to dissolve Matrigel. Organoid structures were then spun down and trypsinized using TrypLE Express (Thermo Fischer Scientific) for 3 min at 37 °C. Following dissociation, cells were washed and stained with PTK7 (188B APC conjugated,1:100, Miltenyi, Paris, France) in 1% BSA in PBS for 30 min on ice. For intracellular staining of organoids, PTK7 stained cells were washed and permeabilized using the BD cytofix/cytoperm kit (BD Biosciences) as per the manufacturer’s instructions. Cells were stained for BCL-2 (sc-7382 FITC conjugated, 1:20, Santa Cruz Biotechnology) and BCL-XL (2764, 1:100, Cell Signaling Technology, Leiden, The Netherlands) for 30 min on ice. BCL-XL staining was analyzed with Alexa Fluor-594 conjugated anti-rabbit secondary antibody (Thermo Fischer Scientific). Both BCL-2 and BCL-XL antibodies were validated on Co01 cells overexpressing the respective proteins (Supplementary Fig.5e–g). All flow cytometry analyses were performed on the FACSCanto (BD biosciences) and PTK7 sorting of *APC*^*KO*^ organoids was performed on the SH800S Cell Sorter (Sony, Hoofdorp, The Netherlands). All stainings were performed as three independent experiments.

### RNA and microRNA isolation and quantitative real-time PCR

Organoids cultures were collected for RNA extraction using cell recovery solution to dissolve the Matrigel, following which the cells were pelleted and RNA was extracted using the NucleoSpin RNA II kit (Macherey-Nagel, Leiden, The Netherlands). For isolation of microRNA, Matrigel was dissolved in cell recovery solution and organoids were pelleted and resuspended in TRIzol (Thermo Fischer Scientific). Chloroform was added and the mixture was centrifuged as per the manufacturer’s instructions. The aqueous phase was carefully collected in a new tube, mixed with 100% ethanol (1.5x volume of aqueous phase) and RNA was isolated using the NucleoSpin RNA II kit (Macherey-Nagel) following manufacturer’s instructions.

For gene expression analysis using quantitative real-time PCR (qRT-PCR), RNA was reverse transcribed to cDNA using Superscript III as per the manufacturer’s instructions (Thermo Fischer Scientific). SYBR green (Roche, Woerden, The Netherlands) was used to perform qRT-PCR following the manufacturer’s instructions on a Roche Light Cycler 480 II. All obtained values were normalized to the expression of *RPLP0* and all primer sequences are provided in supplementary Table 2. For analysis of microRNA expression levels using qRT-PCR, RNA was reverse transcribed to cDNA using the reverse transcription enzyme of the miRCURY LNA RT kit (Qiagen, Hilden, Germany) according to the manufacturer’s instructions. The miRCURY SYBR Green PCR kit (Qiagen) was used to perform qRT-PCR with the following miRCURY LNA miRNA PCR assays: hsa-mir-17-5p and hsa-mir-18a-3p, as per the manufacturer’s instructions (Qiagen). Data shown have been normalized to the expression of U6 snRNA.

### Immunohistochemistry

Matrigel cultures were embedded in paraffin as previously described [58]. Briefly, organoids in Matrigel were fixed with 4% paraformaldehyde (PFA, Klinipath, Duiven, The Netherlands) over night at 4 °C. PFA was then replaced with 70% ethanol for 30 min following which dehydration was carried out with 100% ethanol and then xylene, both twice for 30 min at RT. After dehydration, the Matrigel pieces were incubated in paraffin at 60 °C twice for 30 min and finally embedded in paraffin. Four micrometer sections were cut from paraffin embedded blocks using the microtome. Slides were stained for cleaved caspase-3 (1:200, 9661, Cell Signaling Technology), BCL-2 (15071 S, 1:100, Cell Signaling Technology), and BCL-XL (2764, 1:500, Cell Signaling Technology) using standard procedures according to the manufacturer’s protocols.

### Immunobloting

Analysis of protein expression was done as previously described [45], organoids were collected in cell recovery solution (BD Biosciences) at 4 °C for 30 min to dissolve Matrigel and then lysed using 1x RIPA Lysis and Extraction buffer (Thermo Fischer Scientific) containing Halt protease and phosphatase inhibitor cocktail (1:100, Thermo Fischer Scientific). Protein samples were quantified using the Pierce BCA protein assay kit (Thermo Fischer Scientific) as per the manufacturer’s instructions. About 15 µg protein was loaded per well into 4–15% precast gels (Bio-Rad, Lunteren, The Netherlands) and then transferred to PVDF membranes using the Trans-Blot Turbo transfer system (Bio-Rad) according to the manufacturer’s instructions using the mixed molecular weight transfer settings. Membranes were blocked for 1 h in 5% bovine serum albumin (BSA) in Tris-buffered saline and Tween-20 (TBS-T,1x) and stained with primary antibody overnight at 4 °C. The following primary antibodies were tested: MCL1 (1:1000, #4572, Cell Signaling), BCL-XL (1:1000, #2764, Cell Signaling), BIM (1:500, ADI-AAP-330-E, Enzo life sciences), c-Myc (1:1000, #9402, Cell Signaling), BAX (1:1000, #2774, Cell Signaling), BAK (1:1000, #3814, Cell Signaling), and BID (1:1000, #2002, Cell Signaling), all diluted in 5% BSA in TBS-T. After washing the blots four times for 20 min each with TBS-T, the secondary antibody anti-rabbit-horseradish peroxidase (1:5000, #4050-05, Southern Biotech, Uden, The Netherlands) or anti-mouse-horseradish peroxidase (1:5000, #1031-05, Southern Biotech) was added for 2 h at room temperature. Following another round of 4 × 20 min washes, the membranes were developed using the LumiLight western blotting substrate (Sigma-Aldrich) and imaged on the ImageQuanti LAS4000 (GE Healthcare Life Sciences, Rijswijk, The Netherlands). GAPDH was used as a loading control, a representative GAPDH is shown as multiple blots were run at the same time for proteins of the same molecular weights (1:5000, MAB374, Sigma-Aldrich).

### Cell viability assay

Tumor organoids were isolated using cell recovery solution (BD Biosciences) at 4 °C for 30 min to dissolve Matrigel. Organoid structures were then spun down and trypsinized using TrypLE Express (Thermo Fischer Scientific) for 3 min at 37 °C. Following dissociation, cells were washed, counted, and seeded in 96-well plates at a density of 4000 cells per well in 6 µL Matrigel drops. After overnight incubation, cells were treated in a titration with BH3 mimetics and/or Oxaliplatin using the HP D300 digital dispenser (Hewlett-Packard, Amstelveen, The Netherlands) for 5 days. Organoids were imaged using the EVOS cell imaging system (Thermo Fischer Scientific) and cell viability was assessed using CellTiter-Blue (Promega, Leiden, The Netherlands) according to the manufacturer’s instructions. All viability assays were performed as two or three independent experiments. Synergy was calculated using the Synergy Finder web tool [60] and the Bliss independence model was employed to calculate the Bliss score for the most synergistic three-by-three dose window in the dose-response matrices, positive bliss scores are considered indicative of synergistic interactions.

### Animal experiments

All experiments were performed according to UK Home Office regulations (Project Licenses 70/8646 and 70/9112), adhered to ARRIVE guidelines and were reviewed by local animal welfare and the ethical review committee at the University of Glasgow. Intracolonic inductions in 12–20-week-old male and female *Villin*Cre^ER^*Apc*^fl/fl^ C57BL/6 mice were performed under general anesthesia wherein a single 70 µl 100 µM dose of 4-hydroxy tamoxifen (H7904-5MG from Sigma) was injected into the colonic submucosa via a colonoscope (Karl Storz TELE PACK VET X LED endoscopic video unit). Mice were treated with ABT-199 (*n* = 4), A-1155463 (*n* = 5), or vehicle-only (*n* = 6) at a dose of 100 mg/kg by oral gavage. Drugs were dissolved in 60% Phosal 50PG (Lipoid, Ludwigshafen, Germany), 30% polyethylene glycol 400, and 10% ethanol. For the transformation experiment, treatment was started 2 days prior to tamoxifen induction and continued afterwards on every other day for 28 days. To assess efficacy in preexisting tumors, colonic adenomas were allowed to grow out for 2 weeks after which mice were treated with either vehicle (*n* = 4) or A-1155463 (*n* = 4), five times a week for 28 days. Tumor growth in the colon was monitored weekly, for 3 weeks by colonoscopy. Tumor size was measured relative to lumen size with ImageJ. For the transformation study, mice were randomly assigned to treatment groups and scoring of tumor burden was blinded. For the post-transformation study, mice were scoped to determine tumor presence and as we detect a spread of tumor sizes at this point, they were equally distributed across treatment groups, tumor burden scoring was blinded. Only mice which were lost due to non-tumor growth related factors were excluded, such as puncture during tamoxifen bolus injection or endoscopic examination related death. This led to the exclusion of one mouse in the ABT-199 treatment group of the transformation study. For mouse sample size calculation we aimed to identify a difference in adenoma growth of 50% or more at a 95% confidence interval. Based on the relatively high variation in adenoma growth from mouse to mouse that was estimated to be around 30%, the power calculation indicated that *n* = 4 per group was sufficient to achieve a power of 80%. In addition, due to potential drop outs due to colon puncture during injection, the final group size of 5 was chosen for the transformation setting. In the case of the therapeutic setting *n* = 4 was used as the groups were assigned after tumor initiation and therefore dropouts due to faulty injections were no longer relevant.

### Data analysis and visualization

BCL-2 and BCL-XL expression graphs were generated from a collection of three public Affymetrix datasets (GSE20916, GSE8671, and GSE4183), which were rma normalized and merged using quantile normalization in *R*. *P* values were calculated using the Mann–Whitney test. RNA-sequencing (RNA-Seq) data from the TCGA cohort was normalized from raw counts to FPKM (log2 transformed).

The single-cell RNA-Seq data and cell type annotations were previously published [30] and available from NCBI GEO with accession GSE132465 and GSE144735. The raw counts per sample (separate tumor vs normal per patient) and major cell types are summarized into the mean and standard error, visualized as forest plots and statistically tested using the R package acdx (https://github.com/pwirapati/acdx). Fold-change and bootstrap *p* value calculation (200 replicates) is based on random-effects gamma GLM model.

Single-cell karyotype sequencing was performed on TA cultures, which were isolated using cell recovery solution at 4 °C for 30 min to dissolve Matrigel. Organoid structures were then spun down and trypsinized using TrypLE Express (Thermo Fischer Scientific) for 3 min at 37 °C. The single cell suspensions were lysed in a nuclei suspension buffer containing 10 µg/ml Hoechst 34580 (Sigma-Aldrich). Nuclei were then sorted into a 384-well plate using the BD FACS Fusion, and stored at −20 °C until further processing for library preparation and sequencing. For library preparation, cells were first lysed with proteinase K (Thermo Fischer Scientific) and the genome was subsequently NlaIII digested, individual cell DNA was labeled with an adapter for library preparation consisting of a cell-specific 8 bp barcode, a 3 bp random molecular barcode (UMI), the 5′ Illumina TruSeq small RNA kit adapter, and a T7 promoter. Cell samples were pooled, in vitro transcribed, and then fragmented and reverse transcribed to cDNA. Illumina sequencing libraries were prepared using the TruSeq small RNA primers (Illumina, Eindhoven, The Netherlands) and sequenced for 1x 75 bp single-end sequencing on the Nextseq500 system. After sequencing the data was trimmed (unique identifiers and adapter flanks) and aligned to genome build “GRCh38.p10”. PCR-duplicate reads were removed and single cell calls were determined using Aneufinder (v1.14) using the Hidden Markov Method and DNAcopy described previously [61].

The Marisa dataset is publically available (GSE39582) and was analyzed using Genomics Analysis and Visualization Platform R2 [62].

## Supplementary information

Supplementary Figure 1

Supplementary Figure 2

Supplementary Figure 3

Supplementary Figure 4

Supplementary Figure 5

Supplementary Figure 6

Supplementary Figure 7

Supplementary Figure 8

Supplementary Table 1

Supplementary Table 2

Supplementary Figures

## Data Availability

The authors declare that all data supporting the findings of this study are available within the article and its Supplementary Information files. Data used to generate Fig. 5a, b, e, f are publically available (GSE20916, GSE8671, GSE4183, GSE132465, and GSE144735), as is the Marisa dataset (GSE39582). The publically available TCGA COAD dataset was used to generate Fig. 6i and supplementary Fig. 5a, b.
