# Supplementary material for: BCL-XL is crucial for progression through the adenoma-to-carcinoma sequence of colorectal cancer
Source: Cell Death Differ. 2021 Jun 11;28(12):3282–96. doi: 10.1038/s41418-021-00816-w (PMC8630104; doi:10.1038/s41418-021-00816-w)
Supplement: Supplementary file 11 — Supplementary Figures [file 41418_2021_816_MOESM11_ESM.docx]

**Supplementary Figure 1. BCL-2 and BCL-XL are essential for ISC survival during transformation.**

a. Graphs depict relative outgrowth of wild type (WT) proximal small intestine (SIP)-derived organoid structures quantified 3 days after passaging, upon treatment with 1µM ABT-199 or A-1155463 in combination with 1 µM Tamoxifen. Each dot represents a replicate (minimal n=7 per condition, n=3 independent experiments), error bars indicate s.e.m.

b. Percentage of activated caspase-3 in the 10% TOP-GFP high Co01 cells after 24 h treatment with 1 µM ABT-199, AZD5991 or A-1155463. Data represents mean ± SD. (n=2 independent experiments). ****p < 0.0001, student’s t-test.

**Supplementary Figure 2. BCL-2 dependence is lost immediately after transformation.**

a, b. Graphs depict relative outgrowth of mouse *Apc^-/-^* organoids quantified 3 days after passaging, upon treatment with indicated doses of (a) ABT-199 or (b) A-1155463. Each dot represents a replicate (minimal n=8 per condition, n=3 independent experiments), error bars indicate s.e.m. * p<0.05, **p<0.01, **** p<0.0001, ordinary one-way ANOVA.

c. qRT-PCR analysis of *BCL-XL* expression in *APC^KO^* organoids transduced with control or *BCL-XL* targeting shRNA, normalized to *RPLP0* expression. Data represents mean ± SD.

d. qRT-PCR analysis of *BCL-XL* expression in *APC^KO^* organoids transduced with control or *BCL-XL* overexpression constructs, normalized to *RPLP0* expression. Data represents mean ± SD.

e. Graphs depict relative outgrowth of human normal colon organoids (*WT2*) quantified 3 days after passaging, upon treatment with 1 µM AZD5991. Each dot represents a replicate (minimal n=15 per condition, n=3 independent experiments), error bars indicate s.e.m.

f. Graphs depict relative outgrowth of human *APC^KO^* organoids quantified 3 days after passaging, upon treatment with indicated doses of AZD5991. Each dot represents a replicate (minimal n=9 per condition, n=3 independent experiments), error bars indicate s.e.m.

g. Relative outgrowth of human mutant organoids of indicated genotypes quantified 3 days after passaging, upon treatment with 3 µM of AZD5991 (n=3 independent experiments). Error bars indicate s.e.m.

h. Relative outgrowth of mouse organoids of indicated genotypes quantified 3 days after passaging, upon treatment with 3 µM of A-1155463 (n=3 independent experiments). Error bars indicate s.e.m. **p<0.01, **** p<0.0001, student’s t-test. AK: *ApcKras*, AKS: *ApcKrasSmad4*, AKSP: *ApcKrasSmad4p53.*

**Supplementary Figure 3. BCL-XL is crucial for stem cell survival in patient-derived tubular adenoma organoids.**

a, b. Graphs depict relative outgrowth of human tubular adenomas TA2 and TA4 quantified 3 days after passaging, upon treatment with indicated doses of (a) ABT-199 or (b) A-1155463. Each dot represents a replicate (minimal n=6 per condition, n=3 independent experiments), error bars indicate s.e.m. * p<0.05, ** p<0.01,***p<0.001, **** p<0.0001, ordinary one-way ANOVA.

c. Cleaved caspase-3 stained human TAs after 24h treatment with 300 nM ABT-199 or A-1155463. Scale bars, 200 µm.

**Supplementary Figure 4. BCL-XL inhibition impairs adenoma outgrowth in vivo.**

a, b. Representative images from a colonoscopy of *Villin*Cre^ER^*Apc*^fl/fl^ mice, one (a) and two (b) weeks after being induced with a single injection of 4-hydroxytamoxifen into the colonic sub-mucosa. ABT-199 and A-1155463 treated mice received 100 mg/kg of drug for two days prior to induction followed by treatment every other day until day 28. Red arrows indicate tumors.

c. Representative images from a colonoscopy of *Villin*Cre^ER^*Apc*^fl/fl^ mice three weeks after treatment with either vehicle or A-1155463. Mice were induced with a single injection of 4-hydroxytamoxifen into the colonic sub-mucosa and treatment began two weeks after induction and continued for 28 days.

d. Quantification of colonic tumor size (normalized to the size of the lumen) of mice described in (c), at pre-treatment and after three weeks of treatment. n = 4 for vehicle and n=4 for A1155463.

e. Graphs depict relative outgrowth of SIP-derived organoid structures quantified 3 days after passaging for the indicated genotype, upon treatment with 1 µM A-1155463, at the time of induction. Each dot represents a replicate (minimal n=6 per condition, n=2 independent experiments), error bars indicate s.e.m. **** p<0.0001, ordinary one-way ANOVA.

**Supplementary Figure 5. BCL-2 expression is decreased upon transformation.**

a, b. mRNA expression of *BCL-2* (a) and *BCL-XL* (b) in the TCGA COAD cohort comparing normal and CRC tumor tissues.

c. Representative images of *APC^KO^* organoids outgrowth 7 days after sorting for PTK7 high and low fractions. An equal number of cells was plated for both conditions.

d. Quantification of *APC^KO^* organoid outgrowth following sorting for PTK7 high and low populations (n=3 independent experiments). **p<0.01, student’s t-test.

e, f. Intracellular FACs staining of (e) BCL-2 and (f) BCL-XL in either control or Co01 cells overexpressing the respective proteins.

g. Immunoblot analysis of BCL-2 and BCL-XL protein levels in Co01 control or BCL-2 and BCL-XL overexpressing cells. GAPDH was used as a loading control.

**Supplementary Figure 6. MiR-17-5p regulates BCL-2 expression in *APC-*mutant organoids.**

a. Single cell karyotype-seq data showing the ploidy in individual cells of TA2 and TA4. Graphs show individual cells (horizontal lines) and colors indicate copy number state for a given chromosome (columns).

b, c. Binding sites of miR-17-5p (b) and miR-18a-3p (c) on the BCL-2 3’UTR as predicted by miRmap.

**Supplementary Figure 7.** **MiR-17-5p regulates BCL-2 expression in *APC-*mutant organoids.**

**a.** BCL-2 Intracellular FACs staining plots of data represented in figure 6h.

**b.** Immunoblot analysis of BIM and BCL-XL protein levels in control and mir-17-5p anti-sense (AS) transduced *APC^KO^* organoids. GAPDH was used as a loading control.

**c.** qRT-PCR analysis of c-Myc expression in *APC^KO^* organoids transduced with either AS-control or AS- mir-17-5p, normalized to *RPLP0* expression. Data represents mean ± SD.

**d.** Immunoblot analysis of c-MYC protein levels in AS-control and AS-mir-17-5p transduced *APC^KO^* organoids. CRC cell line LS180 was used as a positive control. GAPDH was used as a loading control.

**e.** qRT-PCR analysis of indicated BCL-2 family members in human colon organoids of indicated genotypes, normalized to *RPLP0* expression (n=2 independent experiments). Data represents mean ± SD.

f. Immunoblot analysis of indicated BCL-2 family members in human colon organoids of indicated genotypes. GAPDH was used as a loading control.

**Supplementary Figure 8. BCL-XL inhibition is synergistic with oxaliplatin in tumor-derived organoids.**

a. Phase-contrast images of p6T and p9T human CRC organoids treated for 72h with 10µM oxaliplatin alone and in combination with 30nM A-1155463. Scale bars, 250 µm.

b, c. 6 x 7 dose matrices of (b) p6T and (c) p9T human CRC organoids treated with oxaliplatin in combination with A-1155463 for 5 days. % inhibition was calculated from viability data measured by cell titer blue, after normalizing to control. Data are the average of two independent experiments. Bliss synergy scores were calculated for each dose combination and positive scores indicate synergistic effects.

**Supplementary Table 1**. List of microRNAs with binding sites on the *BCL-2* 3’UTR that are highly upregulated in colon adenomas and carcinomas.

**Supplementary Table 2**. List of qPCR primers used in the study.
